# Knowledge, practice, and awareness of stroke warning signs and potential risk factors among future rehabilitation specialists in Saudi Arabia

**DOI:** 10.3389/fmed.2026.1758191

**Published:** 2026-02-17

**Authors:** Alaa M. Albishi, Futun Almutairi, Waleed M. Alshehri, Muneera M. Almurdi, Sami S. Alabdulwahab

**Affiliations:** Department of Rehabilitation Health Sciences, College of Applied Medical Sciences, King Saud University, Riyadh, Saudi Arabia

**Keywords:** knowledge, rehabilitation, risk factors, Saudi Arabia, stroke, warning signs

## Abstract

**Background:**

Stroke remains a leading cause of long-term disability and mortality worldwide, with a growing prevalence in Saudi Arabia. While rehabilitation plays a vital role in post-stroke recovery, improving awareness of stroke risk factors and warning signs is essential for timely recognition and management.

**Objectives:**

This study aimed to evaluate knowledge, awareness, and practices regarding stroke warning signs and risk factors among rehabilitation students.

**Methods:**

A cross-sectional study was conducted using an anonymous online questionnaire distributed to 203 rehabilitation students. The survey assessed demographic data and participants’ knowledge regarding stroke risk factors, symptoms, and management awareness.

**Results:**

The findings indicated a moderate level of knowledge about stroke, with an average score of 9.24 out of 13. Participants most frequently recognized hypertension and smoking as major risk factors and identified key symptoms such as motor and speech difficulties. However, significant gaps were observed in understanding that stroke is a brain disease affecting people of all ages, as well as in knowledge of extracranial clot formation, endovascular management, and post-stroke emotional and personality changes after a stroke.

**Conclusion:**

Although this study assessed knowledge rather than clinical practice or patient outcomes, the results highlight the importance of targeted educational initiatives, integrating comprehensive stroke education modules into rehabilitation curricula, and awareness campaigns to address misconceptions and enhance stroke literacy among future healthcare professionals in Saudi Arabia.

## 1 Introduction

Stroke is a major global health challenge and one of the leading causes of long-term disability worldwide (1). According to the World Health Organization, it is the second leading cause of death globally and a primary contributor to persistent adult disability (2). The worldwide burden of stroke continues to rise due to aging populations and increasing prevalence of hypertension, diabetes, obesity, and unhealthy lifestyle practices (2, 3). In the Middle East, stroke incidence has grown markedly, with notable differences in prevalence and mortality rates among countries (4). In Saudi Arabia, stroke has become a major public health concern, with cases projected to rise by 57%–67% over the next decade (4).

Timely acute management is critical for improving stroke outcomes. Early recognition of symptoms is essential, as prehospital delay, the interval between symptom onset or recognition and arrival at the emergency department, directly impacts access to acute treatments and strongly influences functional outcomes (5, 6). This onset-to-door interval often represents the most prolonged and most variable stage of stroke care, emphasizing the need for rapid symptom detection to ensure prompt emergency response and intervention (6). The well-known principle “time is brain” underscores the extensive neuronal loss that occurs with each minute of untreated ischemia, stressing the need to improve public and healthcare worker stroke awareness to minimize preventable delays (7).

Stroke often results in hemiparesis or paralysis contralateral to the lesion (8, 9), along with impairments in vision, speech, and cognition (10). These deficits may persist for years (11) and substantially diminish functional independence and quality of life (12, 13). The resulting physical, emotional, and socioeconomic burdens affect not only patients but also their families and caregivers (14). The economic impact extends from the immediate need for acute medical care to long-term rehabilitation and support services (15). Intensive rehabilitation plays a central role in promoting motor recovery, neuroplasticity, and improved quality of life after stroke (16–20). Although early rehabilitation has demonstrated benefits for functional outcomes and quality of life (21, 22), stroke prevention is one of the most efficient approaches for lessening stroke’s adversarial wellbeing and economic consequences (23).

Given that some stroke risk factors are modifiable, such as hypertension, diabetes, obesity, and physical inactivity, they can be managed through various interventions and lifestyle modifications (24, 25). Not only are these risk factors frequently seen in individuals who have experienced a stroke, but they also negatively impact functional recovery and can lead to increased rates of hospital readmission and overall mortality (26, 27). Thus, understanding stroke risk factors and warning signals is essential for its prevention (28).

Viewed from a finer lens, rehabilitation therapists are uniquely positioned to engage in stroke prevention, treatment, and long-term management through patient education, risk factor modification, and exercise-based rehabilitation programs (16–23, 28, 29). Evidence indicates that rehabilitation specialists frequently employ non-pharmacological lifestyle and activity-based interventions that support overall health and help reduce the risk of recurrent stroke (30–32). Assessing their knowledge of stroke warning signs and modifiable risk factors is therefore critical, particularly regarding the management of comorbidities commonly encountered during stroke care.

Knowledge of stroke among healthcare professionals, including physical therapy students, is often inadequate, which can negatively affect the quality of care (22, 33–37). Although some studies have examined knowledge, attitudes, and practices related to stroke among nurses and physical therapy practitioners (22, 33), no research has assessed knowledge of stroke risk factors and warning signs among rehabilitation specialist students in Saudi Arabia. Previous findings highlight the importance of education, training, and access to information in shaping clinical beliefs and practice behaviors among licensed rehabilitation therapists (22), underscoring the need to evaluate students’ knowledge within their educational programs. Therefore, this study aims to assess knowledge of stroke, its risk factors, and its warning signs among rehabilitation students in Saudi Arabia.

## 2 Materials and methods

### 2.1 Study design and population

A cross-sectional study was conducted through anonymous distribution using an online survey to explore rehabilitation students at King Saud University (KSU), Riyadh, Saudi Arabia, regarding their knowledge of stroke and its risk factors and warning signals. Students were recruited via an email from the rehabilitation department containing a link to an online questionnaire. All participants signed a written consent form before participating in the study.

### 2.2 Eligibility criteria

A cross-sectional study design assessed the rehabilitation students’ knowledge regarding stroke and its risk factors and warning signals at King Saud University in Riyadh, Saudi Arabia. A convenience sampling approach was used to recruit participants, with all students enrolled in the university’s rehabilitation department invited to participate. The study was approved by the Institutional Review Board of King Saud University (no. E-23-8358).

### 2.3 Data-collection tools and procedure

Only rehabilitation students enrolled at King Saud University in Riyadh, Saudi Arabia, during the study period were eligible to participate. Students were invited to participate in an anonymous online survey using the Google platform. The survey was accessible online from February 7 to April 1, 2024. Invitations were emailed through the rehabilitation department containing a link to an online questionnaire. The research procedures and objectives were explained to all participants, and informed consent was obtained before participation. Informed consent entails details about the participants’ right to withdraw from the study at any time and ensures the confidentiality of the information they are given. To protect participants’ privacy, each participant was assigned a unique code, and the data collection sheets were securely stored in a locked online folder to maintain confidentiality.

### 2.4 Questionnaire development and validation

A self-administered questionnaire was used to collect data from rehabilitation students regarding their general stroke knowledge, with selected items related to ischemic stroke, Stroke risk factors, and warning Signals, adapted from previous studies (34, 38–40). The questionnaire used in this study was developed based on a structured, self-administered online format using the Google Document platform. management. It comprises 24 closed-ended questions categorized into three main sections: 8 questions on demographics and general information, three questions on practice toward stroke and awareness of stroke risk factors and warning signals, and 13 questions regarding knowledge about stroke, its treatment, and its consequences. The items in the questionnaire were designed similarly to those used in previous studies focusing on knowledge regarding stroke and awareness of its risk factors and warning signals assessment. The demographic section of the questionnaire aimed to gather information about participants’ age, gender, level of education, specialty, and sources of information.

Six experts from the Department of Rehabilitation Sciences at King Saud University were selected to assess the questionnaire’s content validity and research experience according to their corresponding level of expertise. The research objective and questionnaire items were distributed with clear instructions to evaluate each item based on clarity and relevance. Recommendations from the experts were incorporated to enhance the questionnaire’s face and content validity. The modified survey achieved satisfactory levels of validity, as indicated by the computed scale-level content validity index based on the average method (S-CVI/Avg) (41–44). To evaluate the survey’s content validity, the experts’ item scores were converted to 1 (if the item relevance score was 3 or 4) or 0 (if the item was given a relevance score of 2 or 1). The scale-level content validity index was computed using the average method (S-CVI/Avg) to assess the scale’s content validity (41, 45, 46). To attain satisfactory content validity, an S-CVI/Avg score of at least 0.83 is required (41). The questionnaire achieved sufficient levels of content validity, with 0.94 and 0.93 for clarity and relevance, respectively, indicating that it is an accurate and reliable tool for measuring knowledge on stroke, its risk factors, and warning signals. The experts provided feedback on the clarity and relevance of each item, and their recommendations were incorporated to enhance the questionnaire’s content validity, which was edited in the final questionnaire form.

### 2.5 Sample size estimation

Our sample size was estimated based on previous research, which suggests that a sample size of 100 subjects is recommended for survey research (47, 48). Our sample size was 203, which exceeds 100, ensuring that our study is adequately powered for statistical analysis. This is also considered a large sample size (49, 50).

### 2.6 Data analysis

Statistical analyses were performed using IBM SPSS Software, Version 26. Descriptive statistics summarized the participants’ characteristics, sources of information regarding stroke, general knowledge about stroke, knowledge of stroke risk factors, and knowledge of stroke warning signs. Descriptive statistics were used to summarize the study participants’ characteristics and responses to the survey questions. Categorical variables were reported as frequencies and percentages, while continuous variables were presented as means and standard deviations (SD). The knowledge scores were computed based on correct responses, with one point for each correct answer and zero for incorrect or uncertain responses.

The survey assessed three main dimensions: knowledge about stroke, knowledge of treatment, and consequences of stroke. Each dimension consisted of multiple questions, with responses categorized as correct or incorrect. The frequency and percentage of correct and incorrect answers were calculated for each question to provide an overview of participants’ knowledge. To generate dimension scores, the total score for each dimension was computed by summing the correct responses across all related questions. The overall knowledge score was then obtained by summing the scores of the three dimensions. Mean and SD were calculated for each dimension and for the overall knowledge score to assess the central tendency and variability of participants’ knowledge levels. The knowledge scores were categorized as low, moderate, or high based on Bloom’s cut-off point method (28). The percentage scores were calculated by dividing the raw scores in the “total score” column by the maximum possible score (12) and multiplying by 100 (30). Based on these percentage scores, participants were categorized into three levels of knowledge: Low (≤59%), Moderate (60%–79%), and High (≥80%) (30).

To evaluate differences in knowledge scores across gender, residence area, and awareness of the nearest hospital, independent samples *t*-tests were performed. These tests assessed whether significant differences existed in the mean scores of knowledge about stroke, knowledge about treatment, consequences of stroke, and overall knowledge between the groups. A *p*-value of less than 0.05 was considered statistically significant. For comparisons involving multiple groups, the Kruskal-Wallis H test was used to examine differences in stroke knowledge scores based on marital status, academic level, specialty, and source of information. This non-parametric test was chosen due to the ordinal nature of the variables and the potential for a non-normal distribution of scores. The significance level for all tests was set at α = 0.05.

### 2.7 Ethical considerations

The study was approved by the Institutional Review Board of King Saud University (no. E-23-8358). Before engaging in the online survey, each participant received written informed consent that outlined the study’s objectives and asked for their permission to participate.

## 3 Results

The demographic characteristics of the study participants are presented in Table 1. The mean age was 21.53 ± 2.99 years. The gender distribution was relatively balanced, with males comprising 53.7% and females comprising 46.3% of the sample. Most participants resided in urban areas (89.2%), while only 10.8% lived in rural regions. Regarding marital status, most participants were single (94.6%), with only a small proportion being married (2.5%), divorced (2.0%), or widowed (1.0%). Academic level distribution showed that the largest group was third-year students (36.0%), followed by second-year students (28.6%). The remaining categories- first-year, fourth year, and those with more than 4 years- accounted for 11.8% of the sample.

**TABLE 1:** Demographic information.

Items		Mean ± SD
Age		21.53 ± 2.993
Gender	Male	109 (53.7%)
	Female	94 (46.3%)
Residence area	Urban	181 (89.2%)
	Rural	22 (10.8%)
Marital status	Single	192 (94.6%)
	Married	5 (2.5%)
	Divorced	4 (2.0%)
	Widowed	2 (1.0%)
Academic level	First year	24 (11.8%)
	Second year	58 (28.6%)
	Third year	73 (36.0%)
	Fourth year	24 (11.8%)
Specialty	More than four years	24 (11.8%)
	Physical Therapy	106 (52.2%)
	Occupational Therapy	38 (18.7%)
	Respiratory Therapy	37 (18.2%)
	Speech and Language Therapy	22 (10.8%)
Source of information regarding stroke	Family and friends	37 (18.2%)
	Television	4 (2.0%)
	Social media	29 (14.3%)
	Internet	54 (26.6%)
	Conferences	6 (3.0%)
	Hospital based activities	16 (7.9%)
	Other	57 (28.1%)
Do you know the nearest hospital?	Yes	177 (87.2%)
	No	26 (12.8%)

In terms of specialization, Physical Therapy was the most represented field (52.2%), followed by Occupational Therapy (18.7%), Respiratory Therapy (18.2%), and Speech and Language Therapy (10.8%). Also, regarding our participants’ source of knowledge, we found that the most common source of information was the Internet (26.6%), followed by “Other” sources (28.1%), social media (14.3%), and family and friends (18.2%) were also notable sources. In contrast, traditional media like television (2.0%) and conferences (3.0%) played a minor role. Examining our participants’ awareness of the nearest hospital, we found that 87.2% reported awareness of the location, while 12.8% were unaware.

Examining our participants’ intended responses to stroke symptoms among rehabilitation students. In other words, the practice of immediate action of participants toward anyone showing warning symptoms of stroke (Table 2). When asked about their response to stroke symptoms, the majority (73.9%) chose to contact the ambulance directly and transfer the patient to the nearest hospital. At the same time, a small proportion reported contacting a doctor or family (8.9%), taking aspirin (6.9%), using hot compresses (4.4%), or simply resting (5.9%).

**TABLE 2:** Practice of immediate action of participants toward anyone showing warning symptoms of stroke and their awareness of stroke risk factors and warning signs.

Items		*N* (%)
How would you behave if someone developed symptoms of a stroke	Contact your doctor or family	18 (8.9%)
	Take aspirin directly and wait until symptoms improve	14 (6.9%)
	Massage circularly with hot compresses until symptoms improve	9 (4.4%)
	Take a rest and sleep until symptoms improve	12 (5.9%)
	Contact the ambulance directly and transfer the patient to the nearest hospital	150 (73.9%)
Stroke risk factors	Hypertension	172 (10.6%)
	Diabetes mellitus	81 (5.0%)
	Dyslipidemia	60 (3.7%)
	Smoking	135 (8.3%)
	Elderly age	138 (8.5%)
	Gender (more in males)	81 (5.0%)
	Cardiac disease	134 (8.3%)
	Genetic factors	78 (4.8%)
	Past history of TIA (transient ischemic attack)	104 (6.4%)
	Obesity	132 (8.1%)
	Poor compliance to antiplatelet	53 (3.3%)
	Family history of stroke	79 (4.9%)
	Psychological stress	132 (8.1%)
	Alcohol consumption	115 (7.1%)
	Lack of physical exercise	130 (8.0%)
Stroke warning signs	Speech difficulty	169 (16.5%)
	Focal weakness	122 (11.9%)
	Loss of balance	141 (13.8%)
	Change in consciousness	117 (11.4%)
	Facial asymmetry	123 (12.0%)
	Vertigo	63 (6.2%)
	Visual impairment	89 (8.7%)
	Headache	127 (12.4%)
	Double vision	72 (7.0%)

Given the multiple-choice nature of the questions, the distribution of responses regarding stroke risk factors and warning signs among rehabilitation students are presented in Table 2. In terms of identifying stroke risk factors, the most commonly recognized stroke risk factor was hypertension (10.6%), followed by smoking (8.3%), elderly age (8.5%), cardiac disease (8.3%), and psychological stress (8.1%). However, less emphasis was placed on dyslipidemia (3.7%), poor compliance with antiplatelet therapy (3.3%), and genetic predisposition (4.8%). Notably, alcohol consumption (7.1%) was acknowledged as a risk factor. Our participants also reconditioned lifestyle-related factors, such as obesity (8.1%) and lack of physical exercise (8.0%). Meanwhile, when asked about warning signs, our participants reported that speech difficulty was the most frequently recognized symptom (16.5%), followed by loss of balance (13.8%), headache (12.4%), and facial asymmetry (12.0%). However, less emphasis was placed on vertigo (6.2%), double vision (7.0%), and change in consciousness (11.4%).

Participants’ knowledge about strokes is presented in Table 3, with an overall correct response rate of 62.1% and a mean score of 3.10 ± 1.175 out of 5. One of the most concerning findings is that only 19.2% of participants correctly identified stroke as a disease of the brain, while 80.8% answered incorrectly. Conversely, most participants (76.4%) correctly recognized that stroke is not contagious. The perception of stroke as a condition primarily affecting older individuals was also evident in the responses, with 58.6% correctly rejecting this notion. In comparison, 41.4% still believed that stroke is exclusive to the elderly. Awareness of the hereditary component of stroke was relatively high, with 70.4% recognizing its genetic influence, though nearly 30% remained unaware of this association. Encouragingly, the highest proportion of correct responses was observed for the question on stroke prevention, where 85.7% of participants correctly acknowledged that stroke can be prevented to some extent. The overall level of knowledge was sufficient but remains suboptimal, particularly regarding the nature of stroke as a cerebrovascular disorder.

**TABLE 3:** Knowledge about stroke, its treatment, and its consequences.

Question	*N* (%) of responders		Mean ± SD
	Correct	Incorrect	
1. Stroke is a disease of the brain.	39 (19.2%)	164 (80.8%)	0.192 ± 0.395
2. Stroke is contagious.	155 (76.4%)	48 (23.6%)	0.763 ± 0.426
3. Stroke is an old person’s disease.	119 (58.6%)	84 (41.4%)	0.586 ± 0.494
4. Stroke is a hereditary disease.	143 (70.4%)	60 (29.6%)	0.704 ± 0.457
5. Stroke can be prevented to some extent.	174 (85.7%)	29 (14.3%)	0.857 ± 0.351
Total knowledge about stroke			3.10 ± 1.175
6. Does time have importance in stroke management	158 (77.8%)	45 (22.2%)	0.778 ± 0.416
7. Are you aware of the possibility of clot extraction by endovascular	104 (51.2%)	99 (48.8%)	0.512 ± 0.501
8. Do you know that disability from stroke may improve with timely management	158 (77.8%)	45 (22.2%)	0.778 ± 0.416
Total knowledge of treatment			2.07 ± 0.925
9. Emotional or personality changes can be a consequence of stroke	161 (79.3%)	42 (20.7%)	0.793 ± 0.406
10. Visual problems, e.g., loss of sight, can be a consequence of a stroke	146 (71.9%)	57 (28.1%)	0.719 ± 0.450
11. Cognitive or memory problems can be a consequence of stroke	174 (85.7%)	29 (14.3%)	0.857 ± 0.351
12. Movement or functional problems can be a consequence of a stroke	179 (88.2%)	24 (11.8%)	0.882 ± 0.323
13. Long-term disabilities can be a consequence of stroke	167 (82.3%)	36 (17.7%)	0.823 ± 0.383
Total consequences of stroke			4.07 ± 1.160
Overall knowledge scores			9.24 ± 2.320

Additionally, participants’ knowledge of stroke treatment revealed an overall correct response rate of 69.0% and a mean score of 2.07 ± 0.925 out of 3 (Table 3). Most participants (77.8%) correctly recognized the importance of time in stroke management. A similar proportion (77.8%) also understood that timely management can improve disability outcomes. However, knowledge about endovascular clot extraction was notably lower, with only 51.2% of participants correctly identifying this as a possible treatment, while 48.8% were unaware of this intervention. While rehabilitation students demonstrate a good understanding of the urgency of stroke treatment and the benefits of early intervention, the limited awareness of endovascular clot extraction highlights an area for improvement.

Regarding participants’ knowledge of the consequences of stroke, we found an overall correct response rate of 81.5% and a mean score of 4.07 ± 1.160 out of 5 (Table 3). The highest level of awareness was observed for movement and functional impairments, with 88.2% of participants correctly identifying these. Similarly, cognitive and memory impairments were correctly identified by 85.7% of respondents. Long-term disabilities resulting from stroke were also well-recognized, with 82.3% of participants correctly acknowledging this consequence. Also, emotional and personality changes were correctly identified by 79.3% of respondents. However, nearly 21% did not recognize this as a possible consequence. Visual problems, including loss of sight, were the least recognized consequence, with 71.9% of participants answering correctly and 28.1% incorrectly. Overall, the knowledge score regarding the consequences of strokes was sufficient, suggesting that rehabilitation students have a strong foundational understanding of stroke-related consequences, particularly in terms of motor, cognitive, and long-term disabilities.

An overview of the participants’ total knowledge score related to stroke, encompassing three key dimensions: knowledge about stroke, treatment, and the consequences of stroke. The overall score was calculated as 9.24 ± 2.320 out of a possible maximum of 13, suggesting that, on average, rehabilitation students have a moderate understanding of stroke-related factors.

Statistical analysis examined factors influencing overall knowledge scores and revealed a statistically significant difference between males and females. Males had a mean score of 8.93 ± 2.296, while females had a mean score of 9.62 ± 2.305, with a *t*-value of −2.132 and a *p*-value of 0.034. This statistical difference was mainly driven by females’ knowledge regarding the consequences of stroke. Additionally, the overall knowledge score of strokes was higher among urban participants (9.39 ± 2.235) than rural participants (8.09 ± 2.724), with a *t*-value of 2.505 and a *p*-value of 0.013, indicating a statistically significant difference. While no significant difference was found in basic knowledge about stroke between urban and rural participants, urban students demonstrated significantly better knowledge about stroke consequences and higher overall stroke knowledge. Additionally, the overall knowledge score showed a statistically significant difference, with participants who knew the nearest hospital scoring 9.51 ± 2.078, compared to 7.46 ± 3.049 for those who did not know, with a *t*-value of 3.312 and *p*-value of 0.003.

Moreover, further analysis utilizing the Kruskal-Wallis H tests was used to assess the differences in stroke knowledge (across various dimensions) based on three categorical variables: academic level, specialty, and source of information (Table 4). The results reveal significant differences in stroke knowledge by academic level across all dimensions. For knowledge about stroke (H = 10.858, *p* = 0.028), “knowledge about treatment” (H = 16.764, *p* = 0.002), and “consequences of stroke” (H = 18.682, *p* > 0.001). The overall knowledge score also differed significantly across academic levels (H = 28.839, *p* > 0.001. The “specialty” variable also showed significant differences across several dimensions of stroke knowledge. significant differences were found in knowledge about stroke (H = 8.659, *p* = 0.034), knowledge about treatment (H = 11.215, *p* = 0.011) and “consequences of stroke” (H = 10.035, *p* = 0.018). However, the overall knowledge score (H = 7.830, *p* = 0.050) was not statistically significant. Moreover, the Source of Information variable notably affected knowledge about stroke, with a significant result for all dimensions. The Kruskal-Wallis H test for “knowledge about stroke” (H = 23.070, *p* < 0.001), “knowledge about treatment” (H = 9.736, *p* = 0.136), “consequences of stroke” (H = 10.362, *p* = 0.110), and overall knowledge (H = 24.804, *p* < 0.001).

**TABLE 4:** Factors influencing stroke knowledge.

Items	Knowledge about stroke		knowledge about treatment		Consequences of stroke		Overall knowledge of stroke score	
	Kruskal-Wallis H	*P*-value	Kruskal-Wallis H	*P*-value	Kruskal-Wallis H	*P*-value	Kruskal-Wallis H	*P*-value
Academic level	10.858	0.028	16.764	0.002	18.682	>0.001	28.839	>0.001
Specialty	8.659	0.034	11.215	0.011	10.035	0.018	7.830	0.050
Source of information	23.070	>0.001	9.736	0.136	10.362	0.110	24.804	>0.001

## 4 Discussion

The study utilized a self-administered survey to examine the knowledge, awareness, and practices related to stroke, its risk factors, and warning signs among rehabilitation students in Saudi Arabia. Our study encompasses students from various rehabilitation fields at different academic levels. Physical therapy had the highest representation, followed by occupational therapy, respiratory therapy, and speech and language therapy. This variety highlights a broad range of rehabilitation disciplines (14, 44), which may lead to differences in knowledge and practices regarding stroke and its risk factors, similar to previous studies (14). Additionally, the number of male respondents was slightly higher than that of females, consistent with previous studies (14); a higher proportion of males may reflect that rehabilitation practice in Saudi Arabia is as independent as other medical and health specialties, with a gender distribution discrepancy across all medical fields (51).

### 4.1 Immediate response regarding stroke

In terms of our participants’ *immediate response* (practice) when asked how they would react to stroke symptoms, a small proportion reported incorrect responses, such as contacting a doctor or family, taking aspirin, using hot compresses, or simply resting. These misconceptions suggest gaps in stroke emergency knowledge, emphasizing the need for targeted educational initiatives to reinforce correct stroke response behaviors among future healthcare professionals. Meanwhile, the majority correctly indicated that they would contact an ambulance and transfer the patient to the nearest hospital, aligning with best medical practices for stroke management (14). This may reflect that most participants resided in urban areas. In comparison, fewer lived in rural regions, which may have influenced their access to healthcare services and stroke-related information. Also, more than half of our participants indicated that the Internet, academic coursework, or self-directed learning were the common sources of information, which may highlight the dominant role of digital platforms and coursework in disseminating their health-related responses. Additionally, the majority of participants reported knowing the location of the nearest hospital, a positive indicator of accessibility to emergency care and stroke-related information, similar to previous studies (14), and most participants resided in urban areas. However, those who were unaware could face delays in seeking immediate medical assistance during emergencies, which in turn could impact their response to stroke symptoms.

### 4.2 Awareness of stroke risk factors and warning signs

In terms of identifying stroke risk factors, the most recognized stroke risk factors across our participants were hypertension, followed by smoking, aging, cardiac disease, and psychological stress. These findings suggest that students have a fair understanding of well-established stroke risk factors, particularly hypertension, which is a leading modifiable cause of stroke (7). However, less emphasis was placed on dyslipidemia, poor compliance with antiplatelet therapy, and genetic predisposition. This indicates possible gaps in awareness of these contributors, given that these factors may be out of the scope of the rehabilitation specialists’ knowledge and may be related to other specialized medical fields. Meanwhile, the recognition of multiple lifestyle-related factors, such as obesity and lack of physical exercise (23–25, 28), highlights a growing awareness of the role of these relevant modifiable risk factors in stroke prevention among our rehabilitation specialist students.

On the other hand, among stroke warning signs, speech difficulty was the most frequently recognized symptom, followed by loss of balance, headache, facial asymmetry, and change in consciousness. These findings suggest a reasonable awareness of key stroke symptoms, particularly speech impairment and motor deficits (30–32). These were aligned with our participants’ field of study in different rehabilitation programs where motor and speech problems are hallmark signs of acute stroke problems treated by rehabilitation therapists (16–20, 30–32). However, less emphasis was placed on vertigo and double vision, which can also be critical indicators of posterior circulation strokes, less common than anterior circulation cerebrovascular strokes (52) that require rehabilitation interventions, which may impact the students’ knowledge regarding these symptoms.

### 4.3 Knowledge about stroke, its treatment, and its consequences

Regarding our participants’ knowledge about strokes, the results revealed moderate awareness among rehabilitation students regarding stroke-related concepts. However, specific misconceptions were evident in their responses, highlighting areas where further education may be needed. One of the most concerning findings was that only a small portion of participants correctly identified stroke as a disease of the brain, while the majority answered incorrectly. This suggests a fundamental gap in understanding stroke pathophysiology despite its critical relevance to rehabilitation professionals. Conversely, most participants correctly recognized that stroke is not a contagious disease, demonstrating good awareness in this regard. However, the fact that a noticeable subset of respondents believed stroke to be contagious indicates that some misconceptions persist. The perception of stroke as a condition primarily affecting older individuals was also evident in the responses, with more than half correctly rejecting this notion. In comparison, a considerable proportion still believed that stroke is exclusive to the elderly. Although advanced age is a well-established risk factor, stroke can also occur in younger populations due to genetic predisposition, lifestyle factors, and other medical conditions (53, 54). Awareness of the hereditary component of stroke was relatively high, with most students recognizing its genetic influence, though a considerable proportion remained unaware of this association.

Encouragingly, the highest proportion of correct responses was observed in stroke prevention, recognizing that stroke can be prevented to some extent. This indicates that most students understand the role of modifiable risk factors, such as hypertension, smoking, and physical inactivity, in reducing stroke risk. However, the overall level of knowledge remains below optimal, especially regarding the nature of stroke as a cerebrovascular disorder. These findings underscore the need for targeted educational efforts to address misconceptions and improve stroke knowledge among future rehabilitation professionals.

Meanwhile, participants’ knowledge of stroke treatment showed a generally good understanding of stroke management among rehabilitation students, though some critical gaps still exist. Most participants recognized the importance of time in stroke management, reflecting a strong awareness of “time is brain,” and emphasizing the need for rapid intervention to minimize neurological damage. Similarly, participants understood that timely management can improve disability outcomes, suggesting that most students acknowledge the potential for recovery and functional improvement after acute stroke treatment. These findings indicate a promising level of awareness regarding the urgency of stroke care and the role of early intervention in reducing long-term disability. This is especially important for rehabilitation, considering the well-established evidence supporting early rehabilitation intervention to promote neuroplasticity and functional recovery after stroke (55, 56). However, knowledge about endovascular clot extraction was notably lower, with only about half of the participants recognizing this intervention as a possible treatment option, while a similar proportion were unaware of it. This may reflect that such advanced interventional procedures are often outside the routine scope of rehabilitation specialists’ clinical practice, consistent with findings from previous studies (14). While rehabilitation students demonstrate a good understanding of the urgency of stroke treatment and the benefits of early intervention, their limited awareness of endovascular clot extraction highlights an area for improvement. Improving education on advanced stroke treatments could further enhance their preparedness for clinical practice and help improve patient outcomes in stroke rehabilitation.

Conversely, participants’ knowledge of stroke consequences shows that most rehabilitation students have a strong understanding of the potential long-term effects of stroke, which is essential for their future patient care and recovery roles. The highest awareness was observed for movement and functional impairments, with the majority of participants correctly identifying these as stroke consequences. This is expected, as motor deficits such as hemiparesis and loss of coordination are among the most visible and well-known stroke complications managed by rehabilitation specialists (16–20). Similarly, most respondents recognized cognitive and memory impairments, indicating a solid grasp of the neurocognitive effects of stroke. This awareness is critical as cognitive dysfunction can greatly influence rehabilitation outcomes and the patient’s ability to regain independence. Participants also widely recognized long-term disabilities caused by stroke, reflecting an understanding of the chronic and often disabling nature of stroke, which aligns with core principles of rehabilitation medicine. Most respondents acknowledged emotional and personality changes, demonstrating general awareness of the psychosocial impact of stroke. However, a notable subset did not identify this as a possible consequence, pointing to an area for further education. Given the high rates of post-stroke depression and emotional disturbances (9, 10), it is crucial for future rehabilitation professionals to fully understand the importance of addressing both physical and psychological aspects of recovery. In contrast, visual problems, including vision loss, were the least recognized consequences among participants. While most respondents were aware of these complications, the recognition level was noticeably lower compared to other stroke-related impairments. Since visual deficits such as hemianopia and diplopia can significantly impact a patient’s quality of life and independence (10), incorporating this aspect more prominently in stroke education could be beneficial.

Overall, these findings suggest that rehabilitation students have a strong foundational understanding of stroke-related consequences, particularly in terms of motor, cognitive, and long-term disabilities. However, the lower awareness of emotional and visual complications underscores the need for further emphasis on these aspects in training programs to ensure a more comprehensive approach to stroke rehabilitation. In addition, although students demonstrated awareness of the urgency of stroke management, greater emphasis is needed on key acute treatment principles, including intravenous thrombolysis and the time- and tissue-dependent nature of stroke care, which are essential for optimizing patient outcomes. The aggregation of stroke knowledge scores among our participants was correlated with their source of knowledge, level of education, and specialty, consistent with previous studies (14, 22, 34). Overall, the results indicate that while rehabilitation students have a solid understanding of stroke and its treatment and consequences, there is room for improvement in their knowledge of stroke treatment and some aspects of stroke-related knowledge. Strengthening education in these areas could enhance students’ preparedness for clinical practice and ability to provide high-quality care to stroke patients in rehabilitation settings.

### 4.4 Study limitations and future direction

This study has several limitations. It was conducted at a single institution, King Saud University, which, although recognized as the largest and oldest academic institution in Saudi Arabia, may restrict the generalizability of the findings to other educational or healthcare settings. The use of convenience sampling and voluntary participation may have introduced selection bias, potentially affecting the sample’s representativeness. Additionally, reliance on self-reported knowledge may not fully capture actual clinical competence or behavior. By focusing exclusively on stroke-related knowledge and practices among rehabilitation students, the study may have overlooked other contextual or systemic factors influencing stroke management. Moreover, the questionnaire did not include items addressing the broader role of neurorehabilitation personnel, nor did it assess key acute stroke treatment concepts such as intravenous thrombolysis or the time/tissue window for intervention, which could have provided a more comprehensive evaluation of students’ preparedness for stroke care.

Future research should involve multiple institutions and a more diverse population to enhance external validity and mitigate sampling bias. Longitudinal or interventional study designs are recommended to examine how improvements in stroke knowledge influence clinical decision-making and patient outcomes. Furthermore, integrating simulation-based or practical assessments could provide a more comprehensive understanding of how theoretical knowledge translates into real-world clinical performance.

## 5 Conclusion

This study revealed that rehabilitation students generally possess sufficient knowledge of stroke; however, several important misconceptions remain. Although participants demonstrated awareness of common stroke symptoms, gaps were identified in their understanding of specific risk factors and warning signs, particularly the recognition that stroke is a brain disease that can affect individuals across different age groups and is not solely associated with aging. Limited knowledge was also observed regarding extracranial clot formation, endovascular management, and the emotional and personality changes that may occur after a stroke. While this study assessed knowledge rather than clinical performance, its findings highlight the need to reinforce stroke-related content within rehabilitation education to ensure that future professionals are well-prepared for comprehensive stroke management.

## 6 Recommendations

Targeted educational strategies are recommended to address the specific knowledge gaps identified in this study. Incorporating more comprehensive stroke education modules into rehabilitation curricula, especially those covering stroke pathophysiology, risk factors, advanced treatment options, and post-stroke psychosocial changes, may enhance students’ readiness for clinical practice. Additionally, awareness campaigns promoting early recognition and a timely response to stroke symptoms could further support effective stroke management. Strengthening stroke literacy among rehabilitation students is essential to improving future patient care and reducing the burden of stroke-related disability and mortality in Saudi Arabia.

## Data Availability

The raw data supporting the conclusions of this article will be made available by the authors, without undue reservation.
